# Automatic Recognition of Auditory Brainstem Response Characteristic Waveform Based on Bidirectional Long Short-Term Memory

**DOI:** 10.3389/fmed.2020.613708

**Published:** 2021-01-11

**Authors:** Cheng Chen, Li Zhan, Xiaoxin Pan, Zhiliang Wang, Xiaoyu Guo, Handai Qin, Fen Xiong, Wei Shi, Min Shi, Fei Ji, Qiuju Wang, Ning Yu, Ruoxiu Xiao

**Affiliations:** ^1^School of Computer and Communication Engineering, University of Science & Technology Beijing, Beijing, China; ^2^College of Otolaryngology Head and Neck Surgery, National Clinical Research Center for Otolaryngologic Diseases, Key Lab of Hearing Science, Ministry of Education, Beijing Key Lab of Hearing Impairment for Prevention and Treatment, Chinese PLA General Hospital, Beijing, China; ^3^Institute of Artificial Intelligence, University of Science and Technology Beijing, Beijing, China

**Keywords:** auditory brainstem response, characteristic waveform recognition, neural network model, bi-directional long short-term memory, wavelet transform

## Abstract

**Background:** Auditory brainstem response (ABR) testing is an invasive electrophysiological auditory function test. Its waveforms and threshold can reflect auditory functional changes in the auditory centers in the brainstem and are widely used in the clinic to diagnose dysfunction in hearing. However, identifying its waveforms and threshold is mainly dependent on manual recognition by experimental persons, which could be primarily influenced by individual experiences. This is also a heavy job in clinical practice.

**Methods:** In this work, human ABR was recorded. First, binarization is created to mark 1,024 sampling points accordingly. The selected characteristic area of ABR data is 0–8 ms. The marking area is enlarged to expand feature information and reduce marking error. Second, a bidirectional long short-term memory (BiLSTM) network structure is established to improve relevance of sampling points, and an ABR sampling point classifier is obtained by training. Finally, mark points are obtained through thresholding.

**Results:** The specific structure, related parameters, recognition effect, and noise resistance of the network were explored in 614 sets of ABR clinical data. The results show that the average detection time for each data was 0.05 s, and recognition accuracy reached 92.91%.

**Discussion:** The study proposed an automatic recognition of ABR waveforms by using the BiLSTM-based machine learning technique. The results demonstrated that the proposed methods could reduce recording time and help doctors in making diagnosis, suggesting that the proposed method has the potential to be used in the clinic in the future.

## Introduction

**Figure 1:**
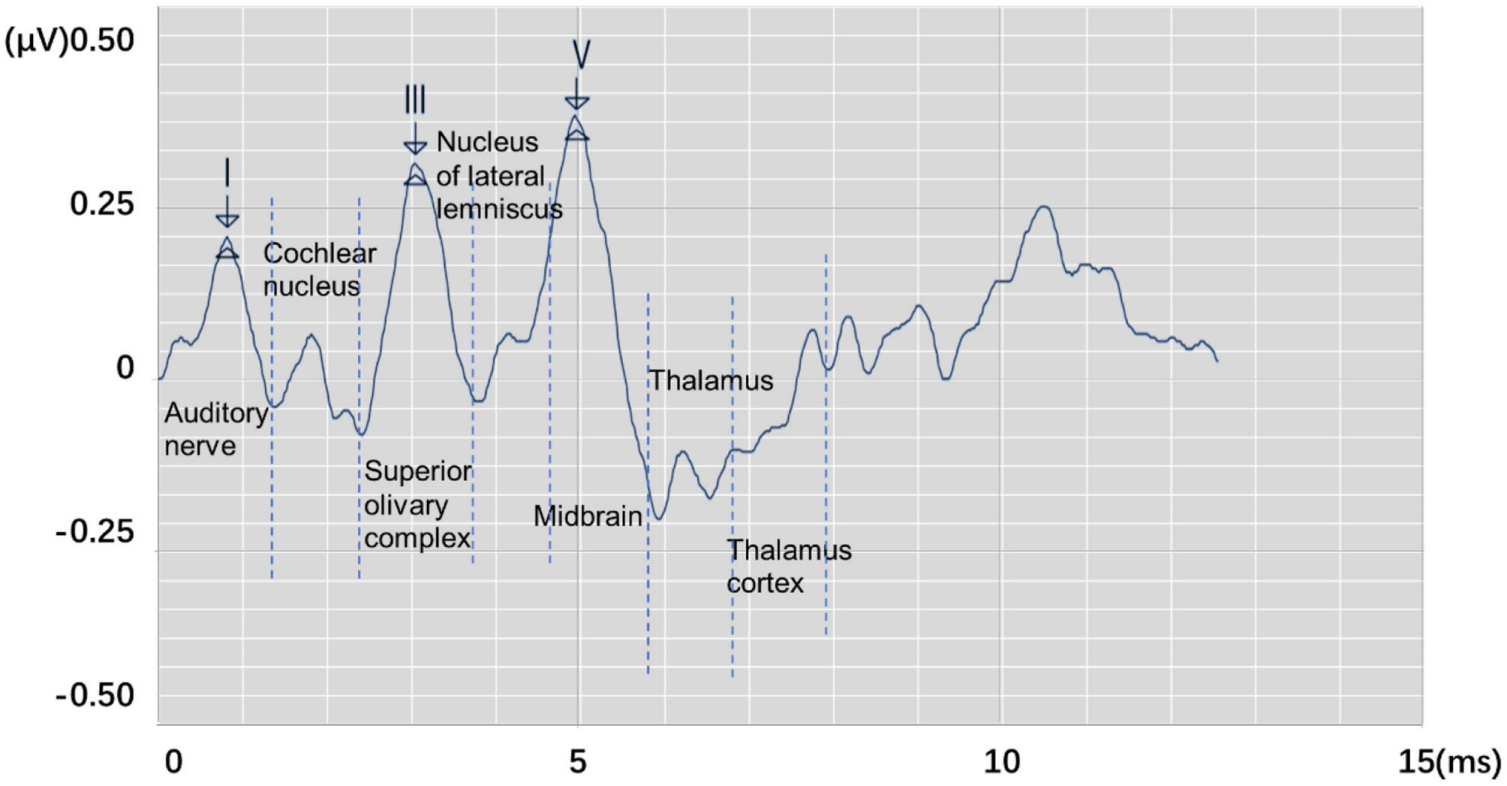
The annotated ABR waveform (legend data is selected from the datasets applied in this work).

Auditory brainstem response (ABR) is a global neural activity in the auditory brainstem centers evoked by acoustic stimulations. It can observe the functional status of the auditory nerve and lower auditory center and reflect the conduction ability of the brainstem auditory pathway (1, 2). Given that patient's hearing impairment can be diagnosed without his active cooperation, ABR has become one of the routine methods for adult hearing recording (3–5). The ABR waveform usually has a range of interwave latency, and its potential in microvolts is recorded. Normal ABR usually has five peaks visible, i.e., waves I, II, III, IV, and V. Wave V usually appears as the largest peak in the ABR. In clinical diagnosis, the minimum intensity of sound stimulation to be capable of evoking a recognized ABR is defined as ABR threshold, which is usually dependent on wave V or wave III (6, 7). Figure 1 shows the annotated ABR waveforms, which are mainly identified as waves I, III, and V clinically. Other characteristic waves are usually not displayed clearly because of small amplitude, two-wave fusion, and noise interference. Thus, they are rarely used as a basis for diagnosis.

In clinical diagnosis, the minimum stimulation intensity of wave V is usually used as ABR threshold. Sometimes, when wave III is greater than wave V, the ABR threshold is judged by stimulation intensity of wave III (8). In determining lesions, the location can be judged according to the interwave latency of waves I, III, and V and the interwave latency between waves and binaural waves (9). Furthermore, the types of deafness of a patient can be judged by observing the change characteristics of ABR waveform latency and the special shape of the ABR waveform in the same patient under different stimulation levels. Thus, the ABR threshold and interwave latency of waves I, III, and V, which are of great significance in clinical applications, can be obtained by identifying the position of the characteristic wave of ABR. Usually, the potential obtained from each stimulation is weak. In a clinical testing, multiple stimulations must be performed to superimpose, average, and obtain relatively stable waveform results. This process is susceptible to interference by electrical noise arising from stray myogenic potentials or movement artifact. In addition, performing multiple tests on patients and comparing results to avoid unobvious peaks, overlapping peaks, and false peaks, which not only consume a lot of time but are also prone to subjective judgment errors, are usually necessary. Thus, identifying the waveform characteristics of ABR and avoiding interference caused by unclear differentiation, fuzzy characteristics, and abnormal waveforms are important issues that need to be solved urgently and correctly in clinical ABR recording.

The application of computer technology in assisting medical diagnosis can effectively reduce errors caused by repetitive work and complex waveform characteristics. This research direction has been important for ABR consultation for a long time (10). For example, Wilson (11) discussed the relationship between ABR and discrete wavelet transform reconstructed waveforms, indicating that the discrete wavelet transform waveform of ABR can be used as an effective time–frequency representation of normal ABR but with certain limitations. Especially in some cases, the reconstructed ABR discrete wavelet transform wave is missing because of the invariance of discrete wavelet transform shift. Bradly and Wilson (12) further studied the method of using derivative wavelet estimation to automatically analyze ABR, which improved the accuracy of the main wave identification to a high level. However, they also mentioned the need for further research on the performance of waveform recognition of abnormal subjects, and manual judgment of abnormal waveforms is still required under clinical conditions. Zhang et al. (13) proposed an ABR classification method that combined wavelet transform and Bayesian network to reduce the number of stimulus repetitions and avoid nerve fatigue of the examinee. Important features are extracted through image thresholding and wavelet transform. Subsequently, features were applied as variables to classify using Bayesian networks. Experimental results show that the ABR data with only 128 repetitive stimulations can achieve an accuracy of 84.17%. Compared with the clinical test that usually requires 2,000 repetitions, the detection efficiency of ABR is improved greatly. However, wave I and wave V are always prolonged by about 0.1 ms and cause wave range changes. Therefore, III–V/I–III would be inaccurate as an indicator.

Thus, automatic recognition of ABR waveforms through computer-assisted methods can assist clinicians and audiologists in ABR interpretation effectively. It also reduces the errors caused by subjective factors, the interference of complex waveforms, and the burden of a large number of repetitive tasks for the medical staff. This study proposes a method of using the long short-term memory (LSTM) network to identify waves I, III, and V in the ABR waveform and proposes a new idea for the recognition of ABR characteristic waveforms by neural networks. The structure of the study is organized as follows: The experimental data and the detailed description of the proposed method are presented in the Materials and Methods section. The Results section presents the experimental design and the corresponding results. Finally, the Discussion section provides an elaboration of the findings of this work.

## Materials and Methods

### Data Source

**Figure 2:**
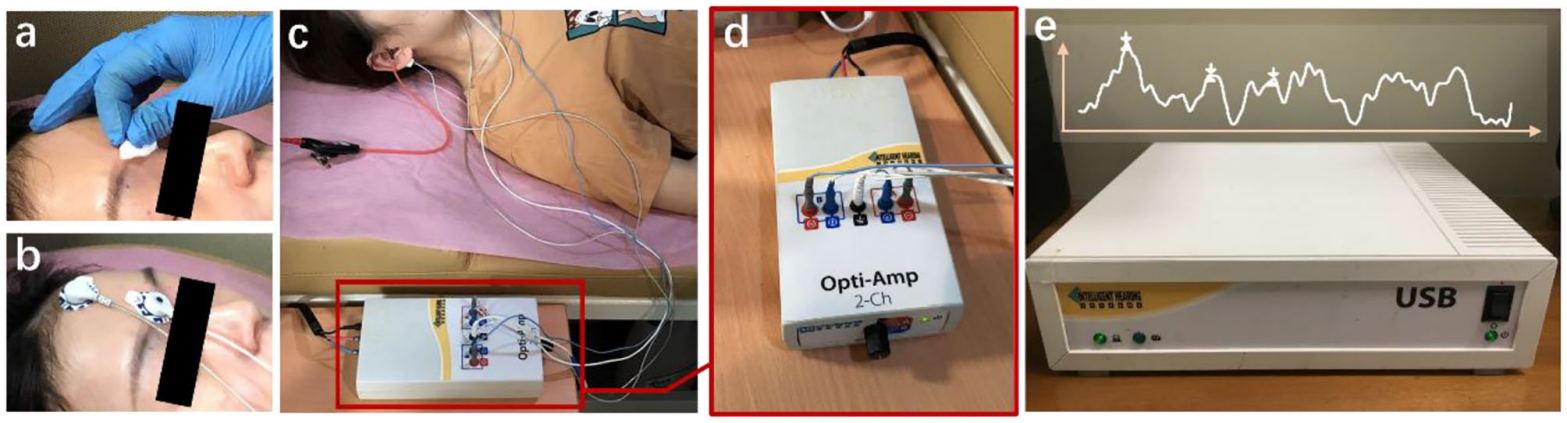
The ABR hearing diagnosis clinical collection process. **(a)** Skin degreasing to enhance conductivity; **(b)** the position of the forehead and earlobe electrodes; **(c)** the positional relationship diagram of the preamplifier, electrodes, and plug-in earphones; and **(d)** the details of the preamplifier. The collected waveform is stored in a server **(e)** and can be observed with the monitor.

The data are provided by the Department of Otolaryngology Head and Neck Surgery, Chinese PLA General Hospital. The SmartEP evoked potential test system developed by the American Smart Listening Company is used for measurement and acquisition. Figure 2 shows the clinical collection process, where Figure 2a represents skin degreasing to enhance conductivity; Figure 2b represents the position of the forehead and earlobe electrodes; Figure 2c represents the positional relationship diagram of the preamplifier, electrodes, and plug-in earphones; and Figure 2d shows the details of the preamplifier. The collected waveform is stored in a server Figure 2e and can be observed with the monitor. Six hundred and fourteen subjects' clinical click stimuli ABR data were collected at 96 dB nHL stimulation intensity after 1,024 repeated stimulations, which contain 181 normal and 433 abnormal hearing. The clinical dataset comprises 348 men and 266 women aged 18 to 90 years old. For data structure, the data contain 1,024 sampling points that range from −12.78 to 12.80 ms with an average interval of 0.025 ms between every two sampling points. All data were marked by three clinical audiologists with characteristic waves: wave I, wave III, and wave V, and cross-validated. Finally, the data were randomly divided into training and test sets. A total of 491 training sets were used to train the network model, and 123 test sets were used for the final recognition accuracy test.

### Data Processing

**Figure 3:**
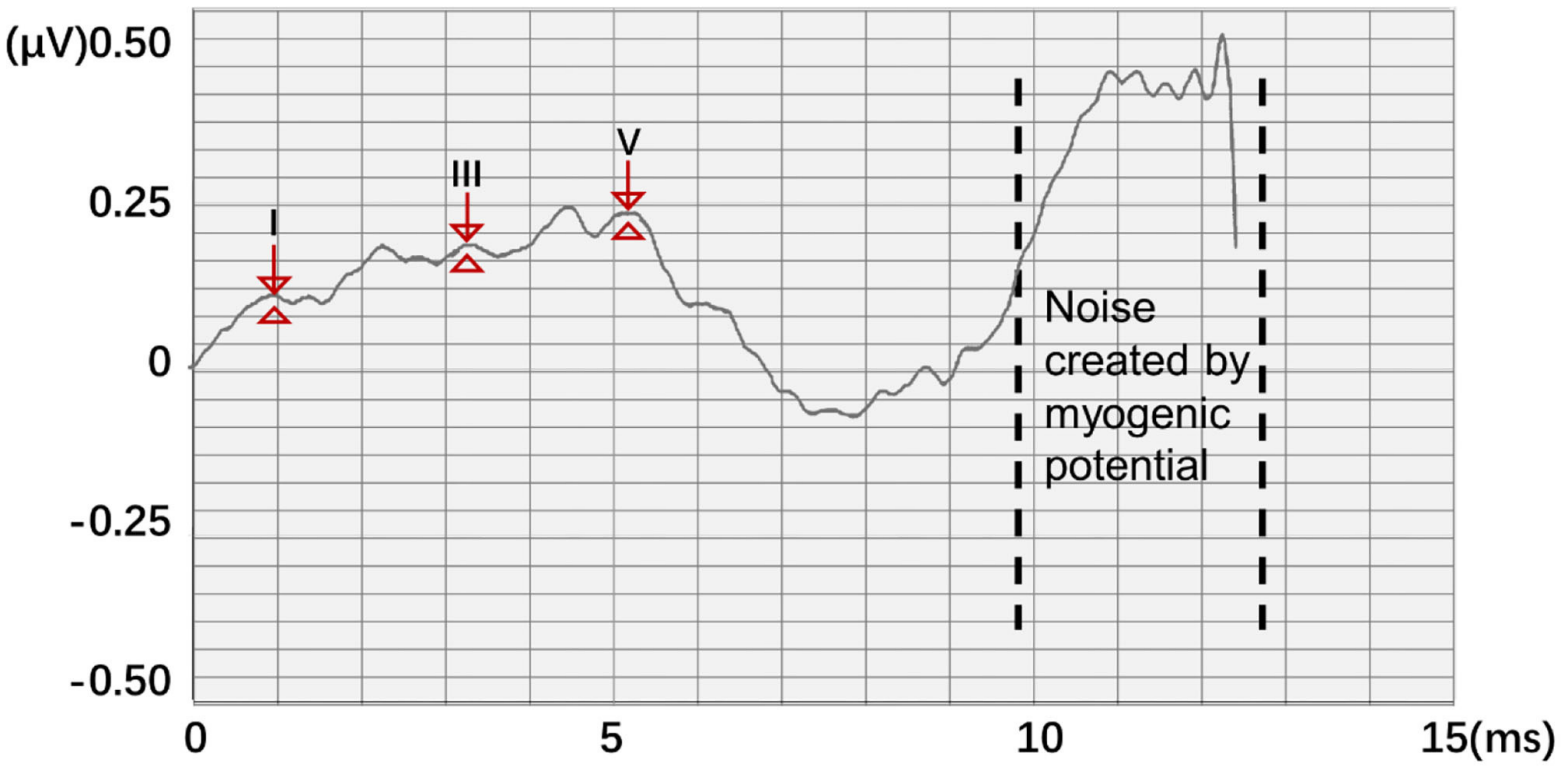
Abnormal ABR waveform and data quantization method.

In this work, a new data processing method is proposed. To quantify waveform and label points, two 1,024 × 1 matrices *A* and *B* were generated as the classification train and label, respectively. *A* represents the potential of the input ABR data. The position of the serial number corresponds to the position of the ABR data sampling point. *B* represents nonfeature (0) and feature points (1), respectively. Thus, according to the position of the label value of the label data, the data that corresponded to the position of the label matrix was changed to 1 to meet the binary classification requirements of all sampling points. However, noise created by myogenic potential is observed in some experimental data (Figure 3). In this ABR clinical test data, the ABR waveform has an unusual increase in the sampling point at the end because of the fluctuation of characteristic waves VI and VII and the result of the external interference. To prevent the interference caused by abnormal data, the data up to 8 ms were selected uniformly to identify the characteristic waves.

On the other hand, the starting point of the actual stimulation is 0 ms. The final potential value input data and the corresponding training label both retained only 321 sampling points of 0–8 ms to avoid interference with neural network training and reduce the amount of calculation in the neural network training process. Thus, *A* and *b*_*f*_ are updated as follows:

(1) $$\{\begin{array}{l} A\left(321\right)={\left\{y_{1},y_{2},...,y_{321}\right\}}^{T}\\ B\left(321\right)={\left\{t_{1},t_{2},...,t_{321}\right\}}^{T} \end{array}$$

In actual processing, the loss function value can easily reach a low level, and sufficient information cannot be learned because the ratio of the labeled value to the unlabeled value in the 321 sample points is only 3:318. The manually labeled information may also bring certain errors. Thus, this study adopted the method of augmenting the position of the identification point in the training label. The four points (0.1 ms) before and after the original marking point were marked as the characteristic area, which expands the marking range of the characteristic waveform.

### Network Structure

LSTM is a recurrent neural network and mainly improved on the basis of the time step unit by adding the output of memory cells to carry information that needs to be transmitted for a long time. Three gate structures are also added. These gate structures are used to select the retention of the memory cell *C*_*t*−1_ value passed from the previous time step, add new information into the memory cell *V*, and predict and output the information transmitted by the memory cell and continue to pass it to the next time step.

**Figure 4:**
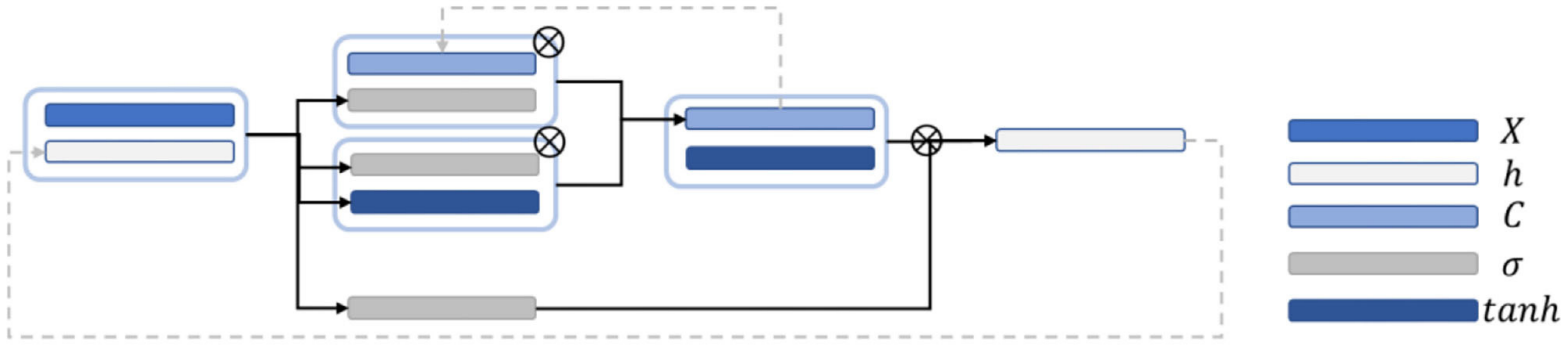
Schematic diagram of the LSTM network structure.

Figure 4 is a schematic diagram of the LSTM structure. First, to control the proportion of the input information retained by the memory cells at the previous time step, the output is calculated as follows:

(2) $$\begin{array}{l} f_{t}=\sigma \left(W_{f}h_{t-1}+U_{f}x_{t}+b_{f}\right) \end{array}$$

*h*_*t*−1_ is the hidden state value passed at the previous time step; and *W*_*f*_, …, and *b*_*f*_ are the corresponding weights and biases. The activation function usually uses the sigmoid function to map the activation value between [0, 1]. To control the proportion of information updated into the memory cell, the sigmoid activation function was first applied to obtain the output *i*_*i*_. Then, the tan*h* activation function is applied to obtain, and the product of the two is used as the information to update the memory cell. *i*_*t*_ and *a*_*t*_ are calculated as follows:

(3) $$\begin{array}{l} i_{t}=\sigma \left(W_{i}h_{t-1}+U_{i}x_{t}+b_{i}\right) \end{array}$$

(4) $$\begin{array}{l} a_{t}=\text{tan}h\left(W_{a}h_{t-1}+U_{a}x_{t}+b_{a}\right) \end{array}$$

where *W*_*i*_, *U*_*i*_, *b*_*i*_, *W*_*a*_, *U*_*a*_, and *b*_*a*_ are the weights and biases. Finally, the memory cell *C*_*t*_ is calculated to the next time step by using Equation (5):

(5) $$\begin{array}{l} C_{t}=C_{t-1}⊙f_{t}+i_{t}⊙a_{t} \end{array}$$

where ⊙ is the Hadamard product, which indicates that the corresponding positions of the matrix are multiplied. The right side refers to the output gate, and the output of the output gate is calculated by using Equation (6):

(6) $$\begin{array}{l} o_{t}=\sigma \left(W_{o}h_{t-1}+U_{o}x_{t}+b_{o}\right) \end{array}$$

where *W*_*o*_, *U*_*o*_, and *b*_*o*_ are the weights and offsets. Finally, the output value *h*_*t*_ at the time step is obtained through using Equation (7):

(7) $$\begin{array}{l} h_{t}=o_{t}⊙\text{tan}h\left(C_{t}\right) \end{array}$$

The predicted output weight and bias are applied to activate the output value to obtain the predicted value, as shown in Equation (8):

(8) $$\begin{array}{l} {\hat{y}}_{t}=\sigma \left(Vh_{t}+c\right) \end{array}$$

Finally, the loss values $\delta_{h}^{t}$ and $\delta_{C}^{t}$ of the hidden state are calculated as follows:

(9) $$\begin{array}{l} \delta_{h}^{t}=V^{T}\left({\hat{y}}_{t}-y_{t}\right)+{\left(\frac{\partial h_{t+1}}{\partial h_{t}}\right)}^{T}\delta_{h}^{t+1} \end{array}$$

(10) $$\begin{array}{l} \delta_{C}^{t}=\delta_{C}^{t+1}⊙f_{t+1}+\delta_{h}^{t}⊙o_{t}⊙\left(1-\text{tan}h^{2}\left(C_{t}\right)\right) \end{array}$$

**Figure 5:**
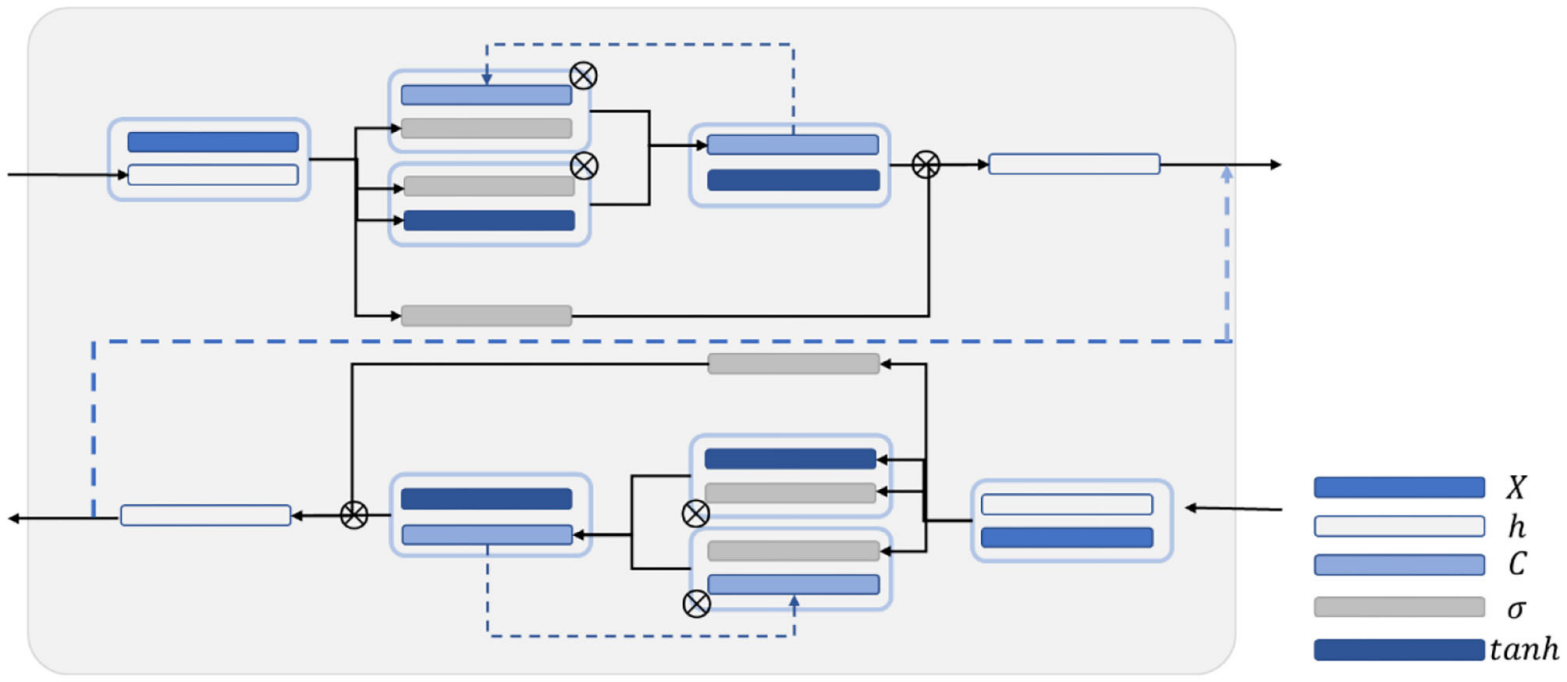
Schematic diagram of the BiLSTM structure.

In this work, BiLSTM is established as the network structure to enable the input sequence to have a bidirectional connection with one another (14). Figure 5 shows that another LSTM layer that propagates backward in time is added on the basis of the unidirectional LSTM forward propagation in time sequence. The final output is determined by the output of the two LSTM layers: forward and backward. Compared with the one-way LSTM, the final output avoids the prediction at each time to only be affected by the input of the previous time. Moreover, it can reflect the information characteristics before and after each prediction point better, thereby making more accurate predictions.

### Wavelet Transform

In the traditional mode, wavelet transform is a commonly used method in ABR extraction and recognition research (15). In ABR extraction, wavelet transform can achieve the effect of eliminating noise by selecting the detailed components of specific frequencies for reconstruction and to make the ABR waveform smoother. Obtaining relatively clear waveforms while reducing repetitive stimulation is also possible. Generally, continuous wavelet transform is defined as (16):

(11) $$\begin{array}{l} WT\left(a,\tau \right)=\frac{1}{\sqrt{a}}\int_{-\infty }^{\infty }f\left(t\right)*\psi \left(\frac{t-\tau }{a}\right)\text{d}t \end{array}$$

where *f*(*t*) is the signal in the time domain, and the part of $\frac{1}{\sqrt{a}}\psi \left(\frac{t-\tau }{a}\right)$ is a wavelet function, which can also be denoted as ψ_*a*,τ_(*t*). Two variables, namely, scale *a* and translation τ, are available. Scale *a* is applied to control the expansion and contraction of the wavelet function, and the translation amount τ controls the translation of the wavelet function. Scale *a* is inversely proportional to its equivalent frequency, which is defined as φ(*t*). The complete wavelet expansion is as follows:

(12) $$\begin{array}{l} f\left(t\right)=\sum_{k=-\infty }^{\infty }c_{k}\varphi \left(t-k\right)+\sum_{k=-\infty }^{\infty }\sum_{j=0}^{\infty }d_{j,k}\psi \left(2^{j}t-k\right) \end{array}$$

where *c* and *d* are the coefficients of the corresponding function, *j* is the frequency domain parameter that determines the frequency characteristics of the wavelet, and *k* is the time domain parameter that controls the position of the wavelet base in the time domain. Although the scale and wavelet functions are complex and have different characteristics, the process of wavelet decomposition can be regarded as using a low-pass filter and a high-pass filter to decompose the signal by frequency. The low-frequency components decomposed in each layer are called approximate components, and the high-frequency components are called detailed components. Thus, approximate components and detailed components were applied to the reconstructed waveform.

## Results

### Experimental Procedure

**Figure 6:**
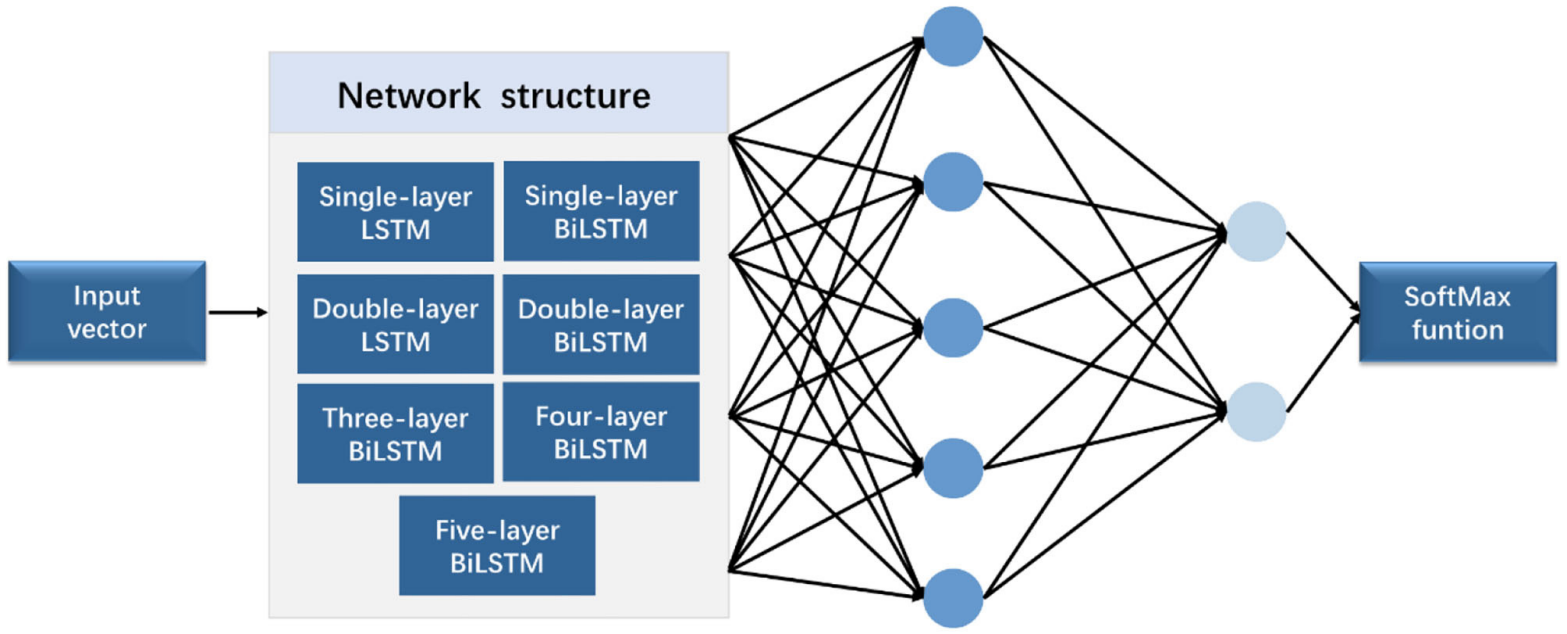
Experimental flowchart.

In this study, three sets of experiments, namely, (1) comparison between various network structures, (2) comparison experiment of wavelet transform, and (3) comparison experiment of different hidden layer nodes, were designed. Figure 6 shows the experimental flowchart. The sequence input layer was used as the input of the potential value of 321 sampling points, and the data were passed to several LSTM or BiLSTM layers. Subsequently, the fully connected layer was connected. The classification probability of each time point was calculated using the softmax function. Finally, the classification layer was connected. The cross-entropy function (17) was used to calculate the loss function of each time point and the overall loss function of the sequence. Then, the time sequence was classified.

In the comparison experiment of multiple network structures, seven network structures, namely, (1) single-layer LSTM, (2) double-layer LSTM, (3) single-layer BiLSTM, (4) double-layer BiLSTM, (5) three-layer BiLSTM, (6) four-layer BiLSTM, and (7) five-layer BiLSTM network layers, were selected. In the comparative experiment of different hidden layer nodes, a three-layer bidirectional LSTM network was used for training, and different numbers of hidden neurons were applied. The experiment applied four groups of different numbers of hidden neurons, namely, 64, 128, 256, and 512.

In the comparative experiment of the wavelet transform, all data added noise as interference. Seven different network structures were used for testing. For instance, the training data preprocessed by wavelet transform were used as the experimental group, and the training data trained using the original data were used as the control group. In this experiment, ABR data were decomposed in six layers, and the approximate and detailed components of the sixth layer and the fourth, fifth, and sixth layers were retained to reconstruct the waveform, respectively. The parameter configuration is consistent. The network was trained with five K-fold cross-validation (*K* = 9), and the test was performed to obtain the average value.

**Figure 7:**
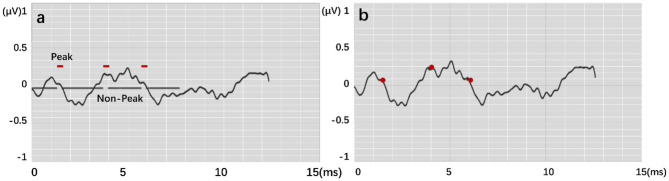
Feature labeling on the ABR, where **(a)** shows output by modes. **(b)** is result by postprocessing.

The output results are in the form of “region.” Figure 7 expresses the output visualization, where the curve is the original ABR used for identification, and the red labels are the network prediction classification results reduced by four times. The ABR of the first 8 ms is clearly divided into two different labels. The part with 1 is the identified peak, and the other part is the identified characteristic nonpeak. Postprocessing is defined as follows: A total of 20 sampling points (0.5 ms) are set as the threshold. The area within 20 sampling points between the beginning and the end is the same characteristic wave area. Finally, the time mean value of the first and last points is calculated as the time value of the recognized characteristic wave. The similar sampling points are calculated to obtain the unique characteristic wave value. Finally, the recognition accuracy rate is calculated according to the identified ABR feature wave position.

**Figure 8:**
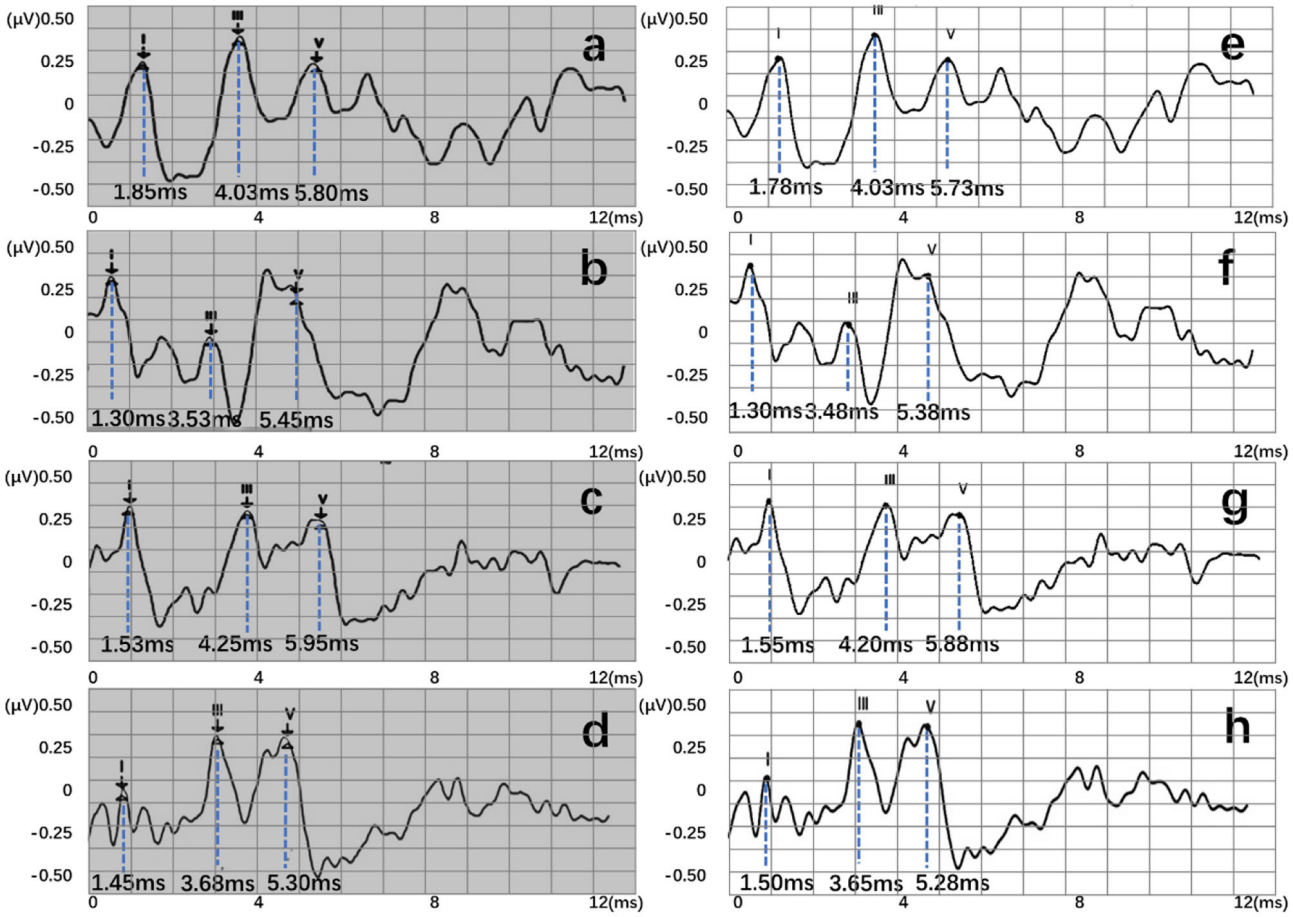
Recognition results of four data, where **(a–d)** are manual labels. Also, **(e–h)** represent outputs of the proposed three-layer BiLSTM model.

**Figure 9:**
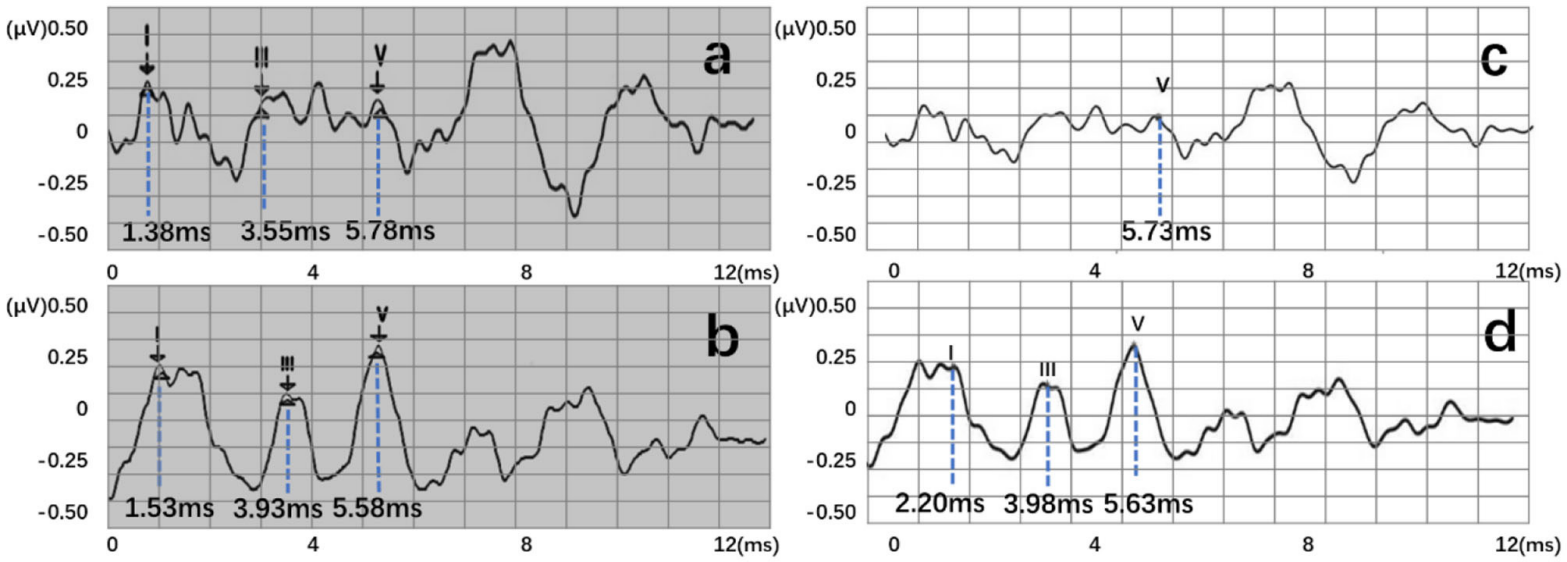
Two error recognition results, where **(a,b)** are manual labels. Also, **(c,d)** represent outputs of the proposed three-layer BiLSTM model.

Four recognition results of ABR data were randomly selected and presented in Figure 8. After postprocessing, output vectors from models were converted to feature points. The identified feature points are almost identical to those selected using manual labeling techniques, illustrating the potential utility of this method in clinical settings. Even in some complex ABR data, manual annotation usually records multiple sets of data to determine the correct peak (Figure 8d). However, the model can directly and accurately identify the peak of the waveform from a single waveform (Figure 8h). Therefore, they also verify the possibility of the proposed method. To better verify the accuracy of recognition, this work has carried out a quantitative discussion from different network structures, wavelet transform processing, and number of hidden neurons. However, the model may also lead to some misjudgments. For example, Figure 9a shows an incorrect recognition result. Since wave I and wave III of the waveform are not obvious, enough continuous identification points cannot be obtained. Therefore, only relatively obvious wave V is obtained after postprocessing (Figure 9c). Also, Figure 9b presents another wrong result. In this case, the obtained error of wave I reached 0.67 ms. This is because the model has judged the wrong wave I (Figure 9d). Thus, in future work, improving the model's ability to analyze complex waveforms is still an important direction.

### Comparison Between Multiple Network Structures

Generally, an error scale of 0.2 ms is applied as a scale range of clinically marked points. Three criterion values for the maximum allowable error value (ME) were tested: −0.1, 0.15, and 0.2 ms. The prediction result was deemed acceptable if the prediction point and the manually identified point were within the ME criteria range. According to the number of correct prediction points *r*_*p*_ and the total marked points *p*_*n*_, the accuracy (ACC) rate is calculated using *r*_*p*_/*p*_*n*_, as shown in Equation (13):

(13) $$\begin{array}{l} \text{ACC}=r_{p}/p_{n} \end{array}$$

In this study, three error scales (ME) of 0.1, 0.15, and 0.2 ms were calculated, respectively, to further explore the recognition accuracy and other related laws. Loss value of training results with different network structures and the ACC under different error scales are revealed in Table 1.

**Table 1:** Loss value and ACC of each network structure.

**Network** **structure**	**Training** **loss**	**Validation** **loss**	**Accuracy** **(0.1 ms) (%)**	**Accuracy** **(0.15 ms) (%)**	**Accuracy** **(0.2 ms) (%)**
LSTM	0.1463	0.1635	37.08	44.92	50.37
LSTMx2	0.1123	0.1625	58.61	65.75	70.59
BiLSTM	0.1264	0.1562	61.96	72.03	77.60
BiLSTMx2	0.0849	0.1285	78.74	84.88	86.84
BiLSTMx3	0.0704	0.1275	85.46	91.06	92.91
BiLSTMx4	0.0651	0.1342	82.48	88.32	90.20
BiLSTMx5	0.0617	0.1467	83.31	88.80	90.90

**Figure 10:**
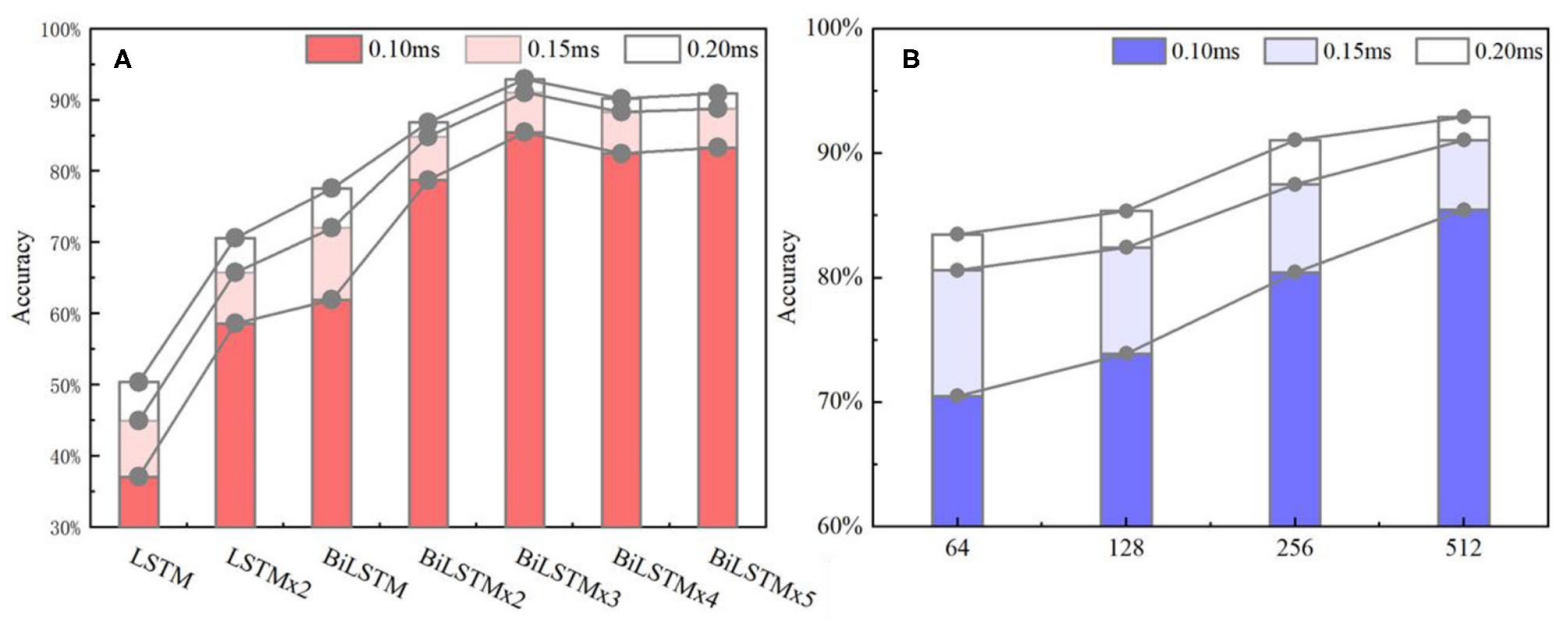
**(A)** ACC metrics with different network structures. In the statistical results, the three-layer BiLSTM network reached 92.91% and is the highest index among all the networks. The single-layer LSTM, which has the lowest index, is about half of it. **(B)** ACC metrics with different hidden nodes, where the 512 nodes ranked first, and the 256 and 128 quantities stood at the second and third positions. Also, the 64 nodes ranked last.

Figure 10A indicates data distribution to observe correlation with different network structures visually. Notably, the ACC of the BiLSTM network is higher than that of the LSTM network. In addition, the ACC of the single-layer BiLSTM network and the double-layer LSTM network is similar. The reason is due to the fact that the two-way LSTM network has a similar structure to the double-layer LSTM network. However, information in the BiLSTM network has the characteristics of propagating in forward and reverse directions, whereas the two-layer LSTM network only propagates in the forward sequence over time. This phenomenon leads to differences in the ACC between the two models. The LSTM and BiLSTM networks increase ACC with the number of superimposed layers. After the BiLSTM network reaches three layers, the ACC will no longer increase significantly. Network structure will gradually reach an over-fitting state and increase computational pressure because of excessive parameters. Thus, the three-layer BiLSTM network is a better choice.

### Wavelet Transform Experiment

**Figure 11:**
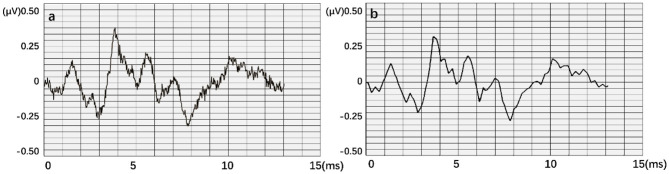
An instance result from the wavelet transform, where **(a)** is the original data. An obvious interference occurred in this waveform. **(b)** is obtained after smoothing.

**Figure 12:**
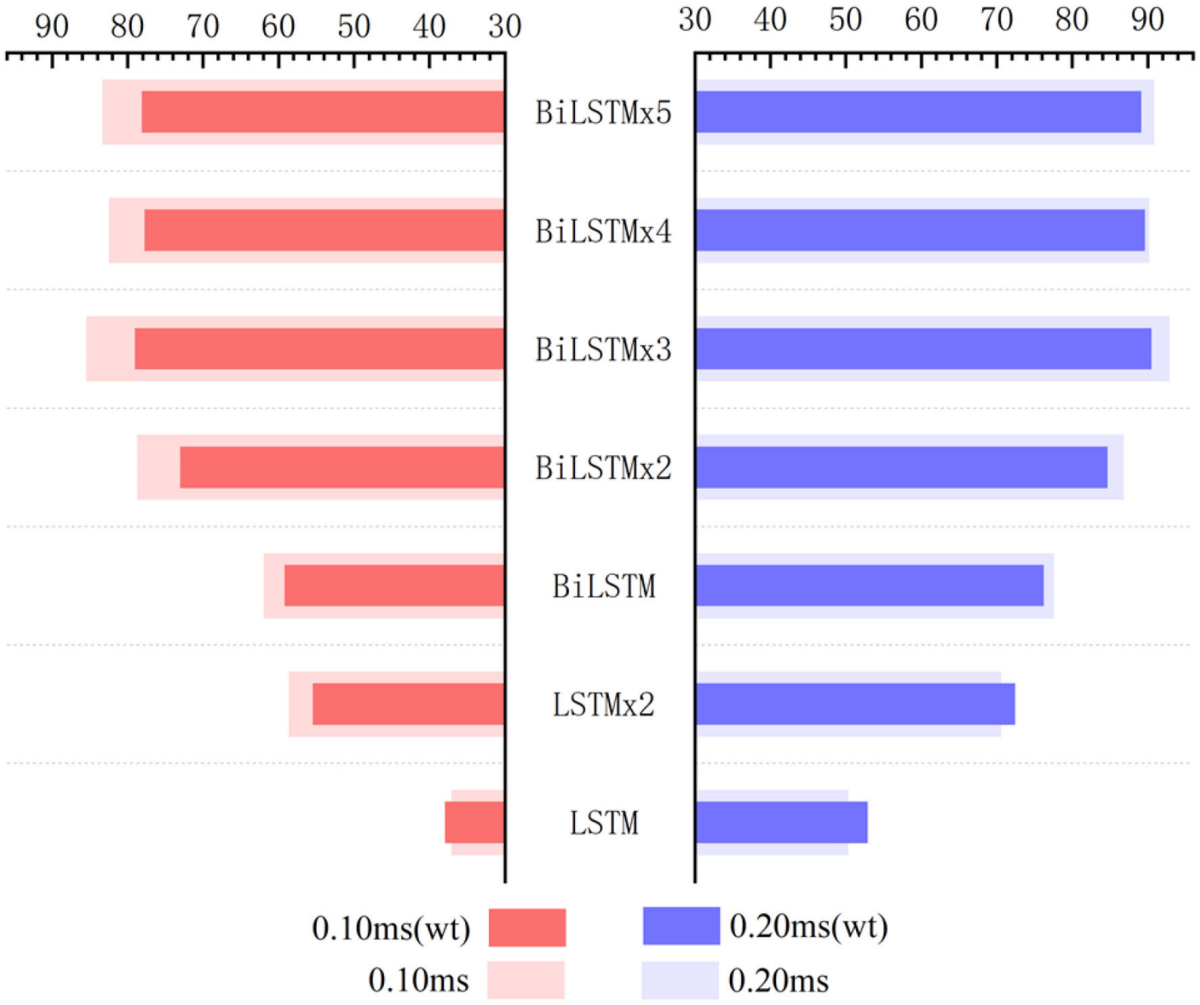
Influence of wavelet transform preprocessing on accuracy. wt represents the results obtained by wavelet transform preprocessing.

When testing the ACC of the wavelet transform, ABR data was decomposed in six layers. Also, approximate components of the sixth layer and detailed components of the fourth, fifth, and sixth layers were retained to reconstruct the waveform. Figure 11 expresses an instance of filtered result by wavelet transform. The curve processed by wavelet transform becomes smoother. Then, unprocessed ABR data served as a control experiment. In this work, detection and comparison were carried out based on two error scales of 0.1 and 0.2 ms (Table 2). The results of recognition ACC are shown in Figure 12.

**Table 2:** The ACC of each network structure with original data and wavelet transform data.

**Network** **structure**	**Original data** **(0.1 ms) (%)**	**Wavelet transform data** **(0.1 ms) (%)**	**Original data** **(0.2 ms) (%)**	**Wavelet transform data** **(0.2 ms) (%)**
LSTM	37.08	37.95	50.37	52.94
LSTMx2	58.61	55.47	70.59	72.46
BiLSTM	61.96	59.17	77.60	76.25
BiLSTMx2	78.74	73.03	86.84	84.71
BiLSTMx3	85.46	79.00	92.91	90.50
BiLSTMx4	82.48	77.73	90.20	89.67
BiLSTMx5	83.31	78.09	90.90	89.17

Recognition ACC values of preprocessing in the LSTM network using wavelet transform are slightly higher than those of the control group. However, they are not as good as those in the control group in the BiLSTM network. Especially, the highest ACC difference reaches 6.46% when calculated with a 0.1-ms error scale. Also, the difference reduces to <3% when calculated with a 0.2-ms error scale. Results indicate that wavelet transform preprocessing does not obtain a higher ACC by smoothing curves. Due to wavelet decomposition and reconstruction, a slight deviation was created in the position of wave crest. Some information was destroyed in the ABR waveform; therefore, the results of training and recognition were affected. This means that the BiLSTM network has noise immunity and can handle low-quality ABR data.

### Comparative Experiments of Different Hidden Layer Nodes

Based on the above results, the three-layer BiLSTM network is a better choice. The ACC results with different hidden node numbers were discussed in this work (Table 3). Figure 10B expresses the ACC results with different hidden layer nodes of 64, 128, 256, and 512. Obviously, recognition ACC increases with the number of hidden nodes, because enough parameters make network fitting accurately. Also, the ACC of the 0.2-ms error scale increases slowly during the change process of 256–512 nodes and is basically saturated. Considering accuracy standard in practical applications and time cost of training that may be brought by the increasing number of hidden nodes, a network of 512 hidden nodes is a better choice.

**Table 3:** The ACC with different hidden layer nodes.

**Hidden layer nodes**	**Accuracy** **(0.1 ms) (%)**	**Accuracy** **(0.15 ms) (%)**	**Accuracy** **(0.2 ms) (%)**
64	70.50	80.61	83.48
128	73.90	82.44	85.36
256	80.44	87.49	91.07
512	85.46	91.06	92.91

Furthermore, this work mainly discusses the characteristic wave recognition process of a click ABR with a 96-dB nHL stimulus. Also, only parameters such as latency and wave interval can be obtained. In clinical applications, many indicators can still be used as a diagnostic basis, such as the relationship between potential values of different stimulus sizes, response and disappearance of wave V, and change of interwave latency of each characteristic wave. This also provides a new idea for the subsequent computer-assisted ABR diagnosis and treatment.

## Discussion

This work proposes an automatic recognition method for ABR characteristic waveforms using the BiLSTM network. The main purpose is to identify positions of characteristic waves I, III, and V, which assist the medical staff in obtaining relevant clinical test parameters, such as interwave latency and wave interval. A data quantification process is designed to analyze the characteristic waveform of ABR, including selection area of potential signal and expansion of label position. An optimal network model structure is obtained through multiple sets of comparative experiments. In 614 sets of clinically collected ABR waveform experiments, the network's overall recognition of characteristic waves showed an ACC of 92.91%.

Experimental results express that the method proposes a new idea for the identification of ABR characteristic waveforms, and helps professionals to obtain interwave latency parameters in ABR waveforms. Therefore, a computer automatic identification method can obtain deeper information, avoid subjective judgment error by the medical staff in the manual identification process effectively, reduce the number of repeated stimulations during a test, and also avoid vision fatigue of the tested person. Because of noise immunity of the proposed network model, it can effectively reduce repetitive detection of patients. In the process of large-scale identification, the average time of each data by using the method only takes approximately 0.05 s, which is much faster than the speed of manual identification. Thus, it has great advantages in repeatable work.

Some efforts have been proposed to analyze ABR waveforms using deep learning methods. For example, Fallata and Dajani (18) proposed a new detection method of ABR based on ANN to reduce detection time. Before ANN calculation, discrete wavelet transform was processed to extract features of ABR. The reduction in recording time was expected to promote the application of this measurement technique in clinical practice. McKearney and MacKinnon (19) divided ABR data into clear response, uncertain, or no response. In their work, they constructed a deep convolutional neural network and fine-tuned it to realize ABR classification. Results showed that the network may have clinical utility in assisting clinicians in waveform classification for the purpose of hearing threshold estimation. Different from the existing works, this research proposed a new data processing method and established an end-to-end deep learning model. The model can also be directly calculated without complicated mathematical transformations, so it provides a new idea for deep learning in signal processing.

## Data Availability Statement

The original contributions presented in the study are included in the article/supplementary material, further inquiries can be directed to the corresponding author/s.

## Ethics Statement

The studies involving human participants were reviewed and approved by The ethic committee of the PLA General Hospital. Written informed consent to participate in this study was provided by the participants' legal guardian/next of kin. Written informed consent was obtained from the individual(s) for the publication of any potentially identifiable images or data included in this article.

## Author Contributions

CC and LZ: conceptualization and writing—original draft preparation. CC: methodology. XP: software and data curation. HQ, FX, and WS: validation. MS: formal analysis. FJ: investigation. QW: resources. RX and NY: writing—review and editing. LZ: visualization. NY: supervision. ZW and XG: project administration. RX: funding acquisition. All authors have read and agreed to the published version of the manuscript.

## Conflict of Interest

The authors declare that the research was conducted in the absence of any commercial or financial relationships that could be construed as a potential conflict of interest.
